# A hospital based retrospective study of factors influencing therapeutic leukapheresis in patients presenting with hyperleukocytic leukaemia

**DOI:** 10.1038/s41598-017-17534-4

**Published:** 2018-01-10

**Authors:** Yanxia Jin, Shishang Guo, Qin Cui, Sichao Chen, Xiaoping Liu, Yongchang Wei, Yunbao Pan, Liang Tang, Tingting Huang, Hui Shen, Guanghui Xu, Xuelan Zuo, Shangqin Liu, Hui Xiao, Fei Chen, Fayun Gong, Fuling Zhou

**Affiliations:** 1 Department of Hematology, Zhongnan Hospital of Wuhan University, Wuhan, Hubei China; 2 Key Laboratory of Artificial Micro- and Nano-Structures of Ministry of Education, School of Physics and Technology, Wuhan University, Wuhan, Hubei China; 3 Key Laboratory of Tumor Biological Behavior of Hubei Province, Wuhan, Hubei China; 4 Department of Laboratory Medicine, Zhongnan Hospital of Wuhan University, Wuhan, Hubei China; 5 Department of Radiation and Medical Oncology, Zhongnan Hospital of Wuhan University, Wuhan, Hubei China; 6 School of Mechanical Engineering, Hubei University of Technology, Wuhan, Hubei China

## Abstract

Therapeutic leukapheresis is a rapid and effective method to reduce early mortality of patients with hyperleukocytic leukaemia (HLL). However, few studies on factors influencing the efficiency have been reported. In this study, 67 cases who underwent leukapheresis were retrospectively analysed and factors related to the collection efficiency of leukapheresis (CE_WBC_) were also evaluated. Paired *t* test showed that there was a significant decrease in statistics of white blood cell (WBC) counts after apheresis. The results of two independent samples nonparametric test suggested that WBC counts, platelet (PLT) counts, haematocrit (HCT), hemoglobin (HGB), serum chlorine (Cl) and globulin (GLB) before leukapheresis correlated with the CE_WBC_. Multiple linear regression analysis with background stepwise variable selection indicated that only WBC and HCT before leukapheresis had an influence on CE_WBC_ significantly. Kaplan-Meier analysis and Cox regression model indicated that lymphocyte (LY) and mean corpuscular hemoglobin (MCH) pre-apheresis as independent factors significantly affected the prognostic survival of patients with HLL. Moreover, platelets and red blood cell were contaminated in the product of leukapheresis. It is an urgent problem to be solved in order to realise higher efficacy and higher purity of WBC collection to improve the survival of patients with HLL through optimising instruments.

## Introduction

Leukaemia is a kind of abnormal haematopoietic stem cell clone of hyperplastic disease. Hyperleukocytosis is one of the high-risk types and defined as peripheral blood leukocyte counts exceeding 50–100 × 10^9^/l^1^. It is characterised by abnormal intravascular leukocyte aggregation and increase of blood viscosity, which results in stasis in the smaller blood vessels^2^. Leukostasis is a poorly understood and life-threatening complication of acute hyperleukocytic leukaemia (HLL), which is a dangerous illness with clinical manifestations of respiratory failure^3^, complicated with intracranial haemorrhage and infarction and other complications^4,5^. The mortality rate is as high as 40% of patients within a week^4,6^. Hyperleukocytosis is present in 5–20% of patients with newly diagnosed acute myeloid leukaemia (AML)^7^. AML with hyperleukocytosis is generally of poor prognosis due to an increased early death rate and a lower response to initial chemotherapy^8–10^.

Leukapheresis is a symptomatic treatment of hyperleukocytosis in acute and chronic leukaemias, and is used to decrease the number of white blood cell quickly for the treatment of patients with HLL in clinic^11,12^, which is the preferred measure in reducing the early mortality of hyperleukocytosis^13,14^. Blood cell separation technology is a kind of simple mechanical removal that can relieve the damage of the leukaemia cells for patients and provide opportunity for late treatment, especially for the elderly patients who cannot tolerate chemotherapy, which is an important measure to reduce early mortality^7,15,16^.

Existing blood cell separators apply the principle of gradient density difference; however, minimal density difference between each blood component (1.6–2.2%)^17–20^, and different blood cells in terms of volume and density has certain degree of overlap, so there are large platelets and red blood cell contaminated in leucocytes in the process of leukapheresis in clinic. A large number of platelets lost by patients in a short time resulted in a significant increased risk of late chemotherapy and delayed the opportunity for better treatment. Therefore, accurate separation of the pure leucocyte is very necessary for HLL patients.

Therapeutic leukapheresis is a rapid and effective means of cytoreduction in HLL patients^15^, and is a safe procedure with regard to organ function and coagulation parameters^11^. Nevertheless, few studies on factors influencing the efficiency have been reported. In the current study, we evaluated the outcomes of 67 newly diagnosed HLL patients that underwent leukapheresis and the effects of leukapheresis on various laboratory parameters. In addition, we investigated the factors influencing therapeutic leukapheresis in patients presenting with hyperleukocytic leukaemia.

## Results

### Clinical characteristics of HLL patients and leukapheresis collection

The study included 67 patients with HLL who underwent therapeutic leukapheresis using the Fresenius COM.TEC instrument in 67 procedures. Clinical characteristics such as age, height, weight, and sex before leukapheresis and blood routine tests are shown in Table 1. The median age was 52 years with 35 males. The blood volume processed was performed by the COM.TEC device. Leukapheresis collection data examined by laboratory is summarised in Table 1. The median WBC yield of 318.00 × 10^9^/l was higher after using the COM.TEC device. However, the collection of RBC counts with mean of 0.60 × 10^12^/l and PLT counts with mean of 271.00 × 10^9^/l were contaminated in the collected product of leukapheresis. The rate of leukocyte depletion (CE_WBC_) was 19.74% which ranges from 0.71% to 109.35%. The additional patient information is shown in Supplementary Table S1 including mortality and (multi) organ failure, which shows that about 40% of HLL patients had pulmonary infection.

**Table 1:** Clinical characteristics of HLL patients and product data. HLL, hyperleukocytic leukaemia.

Item	HLL patients	Median (range)
Clinical characteristics	Age (year)	52.00 (11.00–77.00)
	Height (cm)	160.00 (135.00–180.00)
	Weight (kg)	55.00 (30.00–81.00)
	Sex	35.00 males/32.00 females
Blood routine tests	RBC (×10^12^/l)	2.50 (1.45–4.74)
	WBC (×10^9^/l)	158.00 (58.09–497.50)
	PLT (×10^9^/l)	81.00 (18.00–636.00)
	MNC (×10^9^/l)	21.00 (0.20–135.69)
	LY (×10^9^/l)	19.00 (3.00–279.98)
	NEU (×10^9^/l)	70.10 (19.00–419.68)
	HCT (%)	24.40 (16.40–40.10)
	HGB (g/l)	75.90 (43.30–140.00)
	MCH (pg)	30.90 (19.80–38.10)
	MCV (fl)	94.90 (80.10–156.90)
	MCHC (g/l)	332.70 (175.20–369.30)
Product data	Total blood volume (TBV, ml)	3757.30 (2472.50–5351.20)
	Blood volume processed (BVP, ml)	7440.00 (3049.00–17433.00)
	Product volume (ml)	402.00 (76.00–980.00)
	White blood cell (WBC, ×10^9^/l)	318.00 (12.30–917.88)
	Red blood cell (RBC, ×10^12^/l)	0.60 (0.00–3.82)
	Platelet (PLT, ×10^9^/l)	271.00 (12.00–6476.00)
	**Rate of leukocyte depletion (%)**	19.74 (0.71–109.35)

### Changes of the results of routine blood test and other biochemical characters before and after leukapheresis

**Figure 1:**
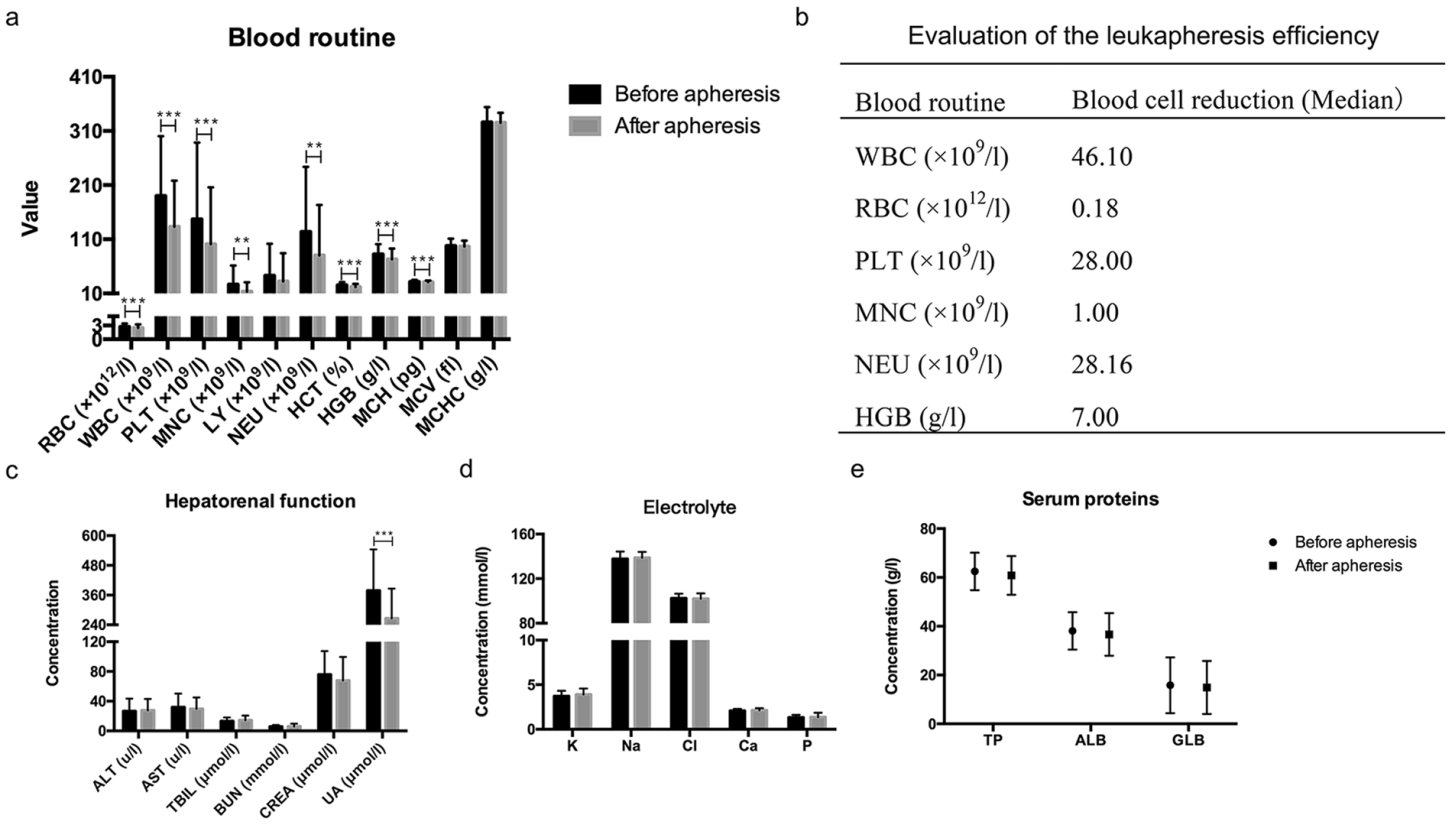
The change of clinical characteristics before and after apheresis. (**a**) blood routine. (**b**) evaluation of the leukapheresis efficiency. (**c**) hepatorenal function. (**d**) electrolyte (serum K, Na, Cl, Ca, and P). (**e**) serum proteins. **p < 0.01, ***p < 0.001. Clinical parameters are shown as Mean ± S.D. RBC, red blood cell; WBC, white blood cell, PLT, platelet; MNC, mononuclear cells; LY, lymphocyte; NEU, neutrophile granulocyte; HCT, haematocrit; HGB, hemoglobin; MCH, mean corpuscular haemoglobin; MCV, mean corpuscular volume; MCHC, mean corpuscular haemoglobin concentration; AST, aspartate transaminase; ALT, alanine aminotransferase; TBIL, total bilirubin; BUN, blood urea nitrogen; CREA, creatinine; UA, uric acid; K, serum kalium; Na, serum sodium; Cl, serum chlorine; Ca, calcium; P, serum phosphorus; TP, total protein; ALB, albumin; GLB, globulin.

The results of paired *t*-test for routine blood tests suggested that red blood cell (RBC) counts (p < 0.001), WBC counts (p < 0.001), PLT counts (p < 0.001), MNC counts (p = 0.008), NEU counts (p = 0.004), HCT (p < 0.001), HGB (p < 0.001), and MCH (p < 0.001) had a significant variation in statistics of leukapheresis (Fig. 1a). The WBC counts were significantly reduced after leukapheresis as shown in Supplementary Fig. S1. The blood cell reduction is shown in Fig. 1b, the median WBC reduction was 46.1 × 10^9^/l, which is accompanied by RBC and PLT decrease. The blood component such as a large number of platelets loss in a short period of time will result in a significant increased risk of late chemotherapy and delay the better opportunity of treatment. The other biochemical parameters included hepatorenal function, electrolytes and serum proteins which were analysed and did not significantly change and almost maintained in normal level after apheresis, especially the abnormal UA levels, which significantly decreased (p < 0.001) (Fig. 1c, d and e). It indicated that leukapheresis had little toxic side effects for organs and did not affect the homoeostasis of the body.

### Factors affecting the rates of leukocyte depletion

**Figure 2:**
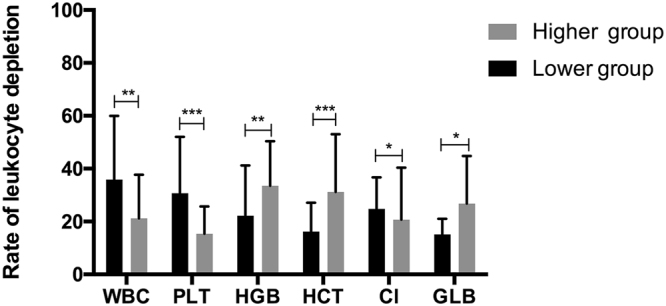
The association between pre-apheresis and rate of leukocyte depletion in HLL patients. Analysed median of each clinical characters, lower than the value as a group, higher than the value for another set. The difference in the rate of leukocyte depletion between two groups was compared with nonparametric test (Mann-Whitney U test) using two independent samples. The cutoff value for WBC pre-apheresis was 100 × 10^9^/l, PLT pre-apheresis was 100 × 10^9^/l, HGB pre-apheresis was 100 g/l, HCT pre-apheresis was 24%. Other variables are grouped using the median as a cut-off value: Cl pre-apheresis (102 mmol/l), GLB pre-apheresis (18.5 g/l).

The median of each biochemical factor are listed in Supplementary Table S2. The results of two independent samples nonparametric test (Mann-Whitney U test) suggested that WBC counts (p = 0.004), PLT counts (p < 0.001), haematocrit (p = 0.001), HGB (p = 0.009), serum chlorine (Cl) (p = 0.029) and GLB (p = 0.034) before leukapheresis were associated with the CE_WBC_ (Fig. 2). The associations between the CEPP and other factors are listed in Supplementary Table S3.

**Figure 3:**
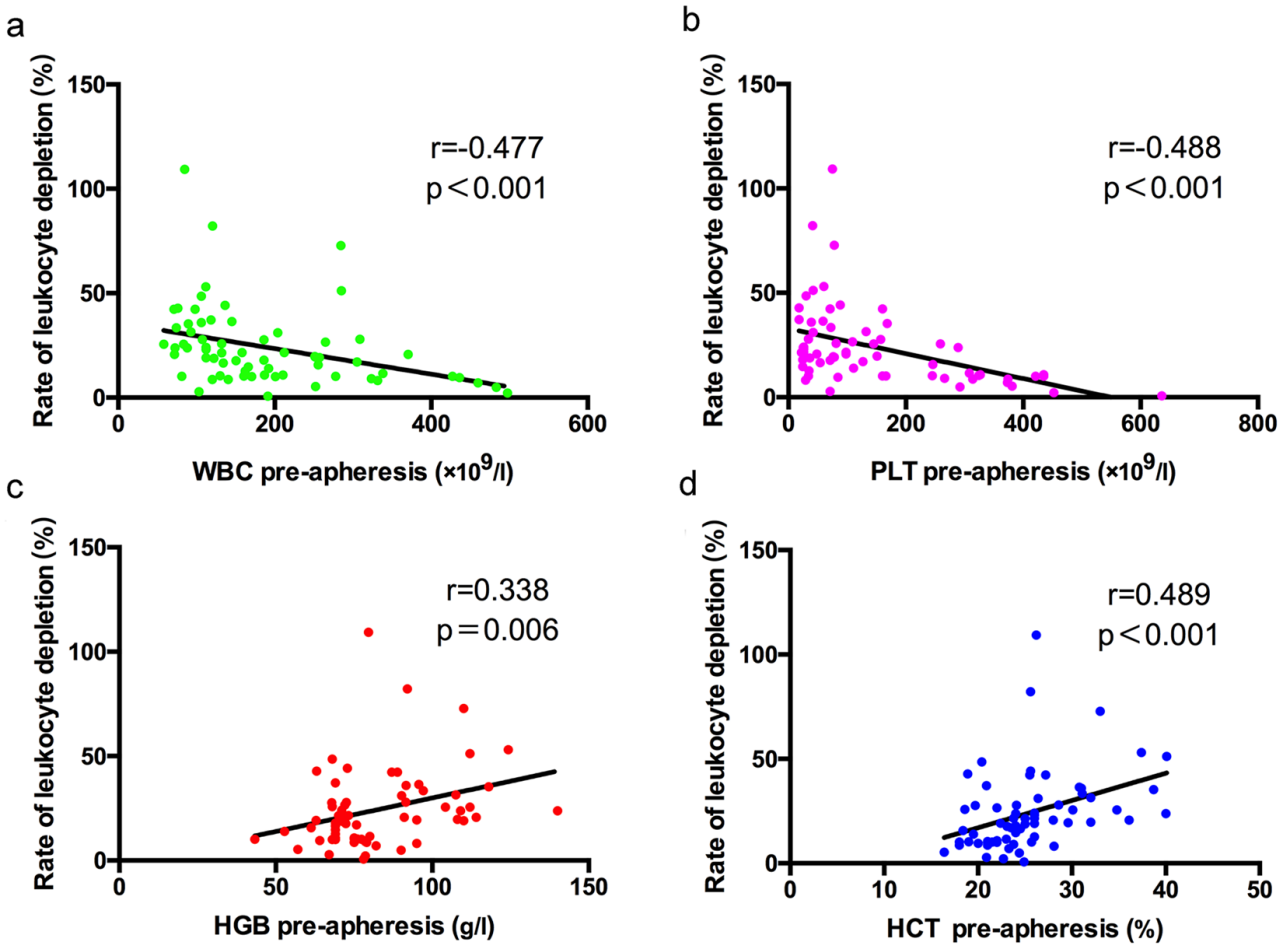
The correlation between pre-apheresis and the rate of leukocyte depletion in HLL Patients. The linear correlation curves of WBC (**a**), PLT (**b**), HGB (**c**) and HCT (**d**). r, correlation coefficient.

Moreover, the correlation between blood routine tests or biochemical factors and CE_WBC_ was analysed with bivariate correlation by nonparametric Spearman test. The results of correlation indicated that CE_WBC_ was negatively correlated with pre-apheresis WBC counts (r = −0.477, p < 0.001), PLT counts (r = −0.488, p < 0.001), NEU counts (r = −0.361, p = 0.022), ALB (r = −0.363, p = 0.014), MCHC (r = −0.276, p = 0.024), serum kalium (K) (r = −0.302, p = 0.035), and serum phosphorus (P) (r = −0.304, p = 0.033). Meanwhile, CE_WBC_ was positively correlated with MNC counts (r = 0.376, p = 0.018), HCT (r = 0.489, p < 0.001), HGB (r = 0.338, p = 0.006), and MCV (r = 0.263, p = 0.031) before leukapheresis (Supplementary Table S4). The linear correlation of CE_WBC_ and WBC pre-apheresis is shown in Fig. 3a, PLT pre-apheresis in Fig. 3b, HGB pre-apheresis in Fig. 3c, HCT pre-apheresis in Fig. 3d. The other clinical characters demonstrated no correlation with CE_WBC_.

In addition, the correlation analysis between the six variables (pre-apheresis WBC counts, PLT counts, MNC counts, HGB, HCT and CE_WBC_) is shown in Supplementary Table S5. Furthermore, the multiple linear regression analysis indicated that WBC counts and HCT before apheresis as independent factors affected the CE_WBC_ (Table 2). The pre-apheresis WBC counts were negatively related with CE_WBC_ and the pre-apheresis HCT was positively correlated with CE_WBC_.

**Table 2:** Multiple linear regression analysis with backward stepwise selection between factors and CE_WBC_. B, regression coefficient.

Item	Unstandardized B	95% CI for B	Standardized Coefficient Beta	t	*p* value
WBC pre-apheresis	−0.046	−0.083~−0.008	−0.323	−2.479	0.018
HCT pre-apheresis	1.313	0.493~2.133	0.433	3.254	0.003

### The survival analysis in leukapheresis

**Figure 4:**
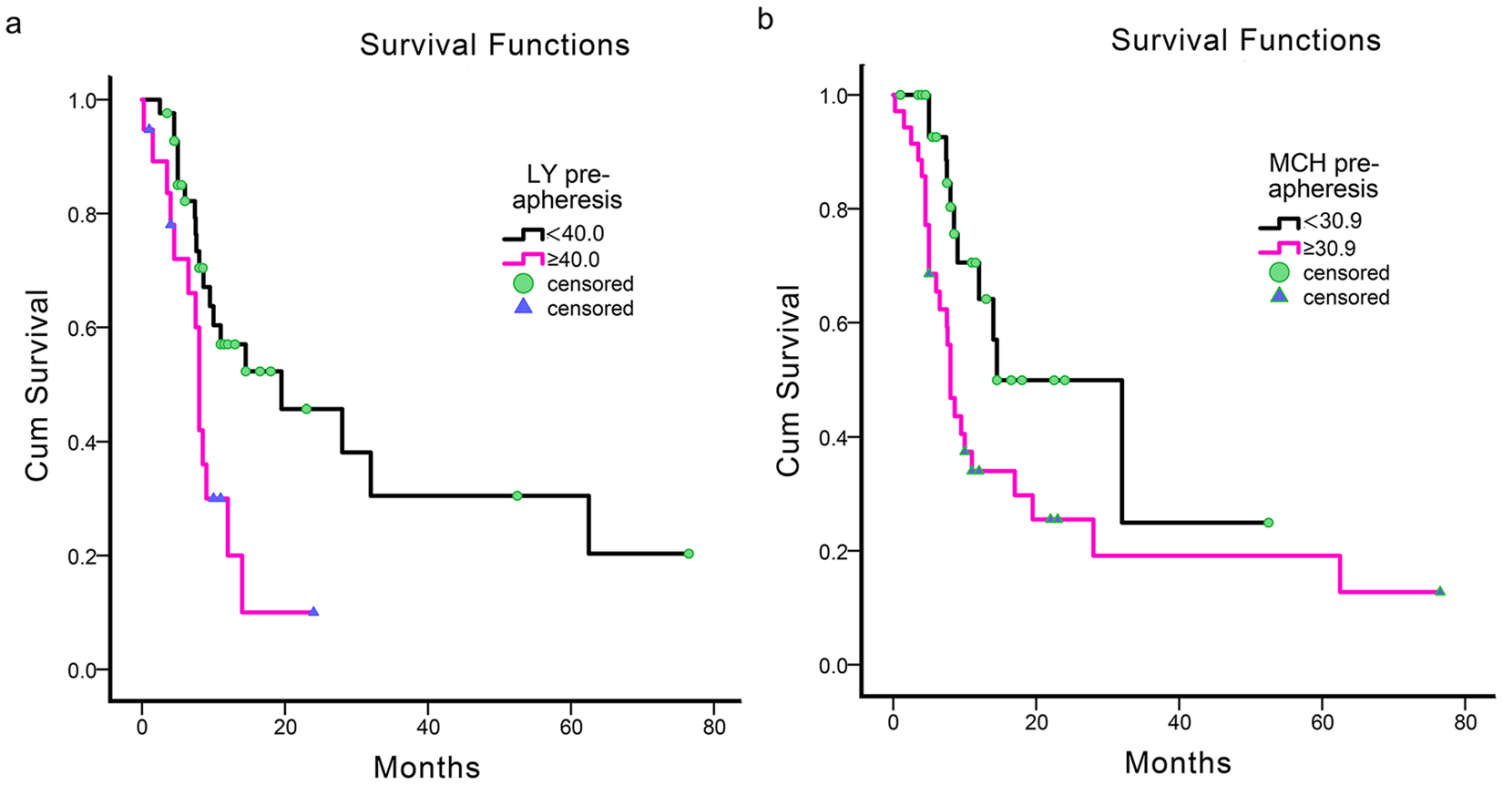
Kaplan-Meier plot analysis of the survival of HLL patients. Survival curves of LY (**a**) and MCH (**b**) pre-apheresis.

In this retrospective cohort study, the overall survival (OS) was analysed by Kaplan-Meier method to evaluate the correlations between the factors before leukapheresis and survival. We found that lymphocyte (LY) counts (p = 0.006), HCT (p = 0.022), and MCH (p = 0.021) significantly affected the survival of HLL patients (Table 3). Cox regression analysis suggested that MCH and LY counts as independent predictors were significantly correlated with the overall survival of HLL patient (Table 4), and the survival curves are shown in Fig. 4. The HLL patients with MCH ≥ 30.9 pg pre-apheresis and/or LY counts ≥ 40.0 × 10^9^/l will be at a greater risk and poorer survival.

**Table 3:** Kaplan-Meier analysis of the correlation between the leukapheresis and the survival of patient with hyperleukocytosis with the two-sided log-rank test. Months were calculated from leukapheresis to presentation in April 2017.

Variates		Median survival	Logrank	*p* value
LY pre-apheresis	<40.0	8.55 months	7.510	0.006
	≥40.0	8.00 months		
HCT pre-apheresis	<24.0	9.50 months	5.269	0.022
	≥24.0	7.50 months		
MCH pre-apheresis	<30.9	8.50 months	5.362	0.021
	≥30.9	8.00 months

**Table 4:** Cox regression analysis of patient survival in the current retrospective cohort with forward stepwise selection.

Variates	B	*p* value	HR	95% CI
MCH pre-apheresis	1.040	0.007	2.829	1.324–6.045
LY pre-apheresis	1.241	0.001	3.460	1.624–7.374

B, regression coefficient. HR, hazard ratio. CI, confidence interval.

## Discussion

Leukaemia is the most common malignant tumour of the blood, which increases morbidity and mortality and HLL can induce leukostasis, tumour lysis syndrome and disseminated intravascular coagulopathy (DIC) especially in patients with acute leukaemia^9,21^. The major reasons for the early death of HLL patient are tumour-associated nervous and respiratory system invasion, and metabolic disorders caused by tumour lysis^2,21^. The standard treatment for hyperleukocytic leukaemia included leukapheresis, chemotherapy, supporting treatment, and prophylactic treatment for tumour lysis^2^. Therapeutic leukapheresis has an important significance for patients with hyperleukocytic leukaemia, which has been used to achieve more rapid cyto-reduction with the potential to reduce tumour lysis syndrome. These 67 patients did not develop tumour lysis syndrome in our analysis. Leukapheresis can not only decrease the number of peripheral WBC, but also increase S and G_2_/M phase leukaemia cells to improve the sensitivity of chemotherapy for proliferating leukaemia cells^2^. The reports retrospectively analysed AML patients who were treated with leukapheresis, demonstrating that leukapheresis could reduce the early death of HLL patients^1,10,22^, and Bug *et al*. reported that the use of leukapheresis immediately had improved survival within the first 3 weeks of treatment for AML patients with hyperleukocytosis^22^. Nevertheless, no significant correlations were found between leukapheresis and the long-term survival of patients with hyperleukocytic leukaemia^1,13,23^, it did not even improve the 30-day mortality for hyperleukocytic AML patients^24^. Oberoi *et al*. also showed an important meta-analysis demonstrating that leukapheresis does not improve overall survival in HLL patients^25^. In our data, the total survival rate of 44.8% was significantly improved when only one patient among all HLL patients died within a week after apheresis (Supplementary Table S1).

The risk stratification for AML patients is mainly according to the European LeukaemiaNet (ELN) classification for prediction of gene aberrations^26^. Clinically, high leukocytes patients are considered as high-risk patients. Nevertheless, the risk stratification of ELN is based on the cytogenetics, which divides the high leukocytes patients into low-risk, intermediate-risk and high-risk. The survival rate of HLL patients according to ELN classification for AML is shown in Supplementary Fig. S2. There was no significant correlation between leukocyte counts and survival, but leukocyte counts were significantly correlated with ELN classification in our study (p = 0.038, r = 0.254). In addition, Greenwood *et al*. demonstrated that elevated WBC counts only affected the high early mortality and was not related to other prognostic parameters^27^. It is not clear whether that prognostic effect of hyperleukocytosis has different pathophysiology or clinical characteristics. HLL may be an expression of a molecular change, such as the molecular aberration of FLT3-ITD mutation in AML, which has poor prognosis, rather than the actual WBC counts^28^.

The WBC counts pre-apheresis were positively correlated with BUN, CREA and UA before apheresis, so HLL patients are easily prone to renal impairment according to our study. The correlations between clinical parameters pre-apheresis and the presence of symptoms/failures caused by the hyperleukocytosis are listed in Supplementary Table S6. The hepatorenal markers and other parameters have not significantly changed and are almost maintained in normal level after apheresis (Fig. 1c, d and e), which suggested that leukapheresis had little toxicity for organ failures and did not affect the homeostasis of the body compared to chemotherapy. Low damage and rapidly reduced tumour load is an important issue for the treatment of leukaemia. Leukapheresis is just to separate the leukaemia cells from the body, but chemotherapy can lead to normal and malignant cell death including the immune cells with more complications. The risks of delaying start of chemotherapy for HLL patients include rebounding of the WBC counts, tumour lysis syndrome (TLS), disseminated intravascular coagulation (DIC) and even death. These 67 patients did not delay the chemotherapy and did not develop tumour lysis syndrome in our analysis. Alaa M. Ali *et al*. reported that hydroxyurea works more slowly than induction chemotherapy, and the cytoreductive effect should be expected to occur within 24–48 h^4^. Leukapheresis rapidly reduced the WBC counts with more rapid reduction of the leukaemic cellular burden than chemotherapy. After leukapheresis, the WBC counts decreased significantly and the clinical course was significantly improved including renal function (Fig. 1c) and clinical manifestations (Supplementary Table S1). The time of cytoreductive therapy was shortened, and patients started to receive standard regularisation chemotherapy after about a median of two days in our data, so the treatment of regularisation was advanced.

Apheresis system separates blood cells and collects specific blood component by density gradient centrifugation. Our results suggest that the mean rate of leukocyte depletion is 19.74%. Of note, the rate of leukocyte depletion of one patient was more than 100%. The WBC counts decreased after apheresis, but increased only in one patient. It may be that the high leukocyte in HLL patients are both present in the peripheral blood and are deposited in organs such as the spleen. The white blood cells that are deposited in the spleen are also released into the peripheral blood when apheresis, which leads to the increased WBC counts and the depletion will be more than 100%. Sarkodee-Adoo *et al*. also reported cases where the rate of blood cell depletion was more than 100%^29^. In the present study, the median rate of WBC reduction was 28.4%. Holig K demonstrated that WBC counts could decrease by 10%-70% with one cycle of leukapheresis^12^. Bruserud *et al*. conducted 35 leukapheresis for 16 patients with AML and the associated rate of WBC reduction was 48.3%^15^. Yilmaz *et al*. conducted 29 leukapheresis for 12 children diagnosed with acute leukaemia, and associated rate of WBC reduction was 36%^30^.

Among the 67 leukapheresis, our results of one-variable linear correlation and regression suggested that WBC counts before leukapheresis were negatively correlated with the rate of leukocyte depletion, and significant correlation was found between pre-apheresis WBC counts and the rate of leukocyte depletion (p = 0.018). The higher counts of WBC were associated with lower rate of leukocyte depletion, which could be explained by the fact that the increase of leukostasis made blood viscosity increased, blood flow velocity decreased, and the processed blood volume decreased, and then the rate of leukocyte depletion decreased. Meanwhile, our study also indicated that haematocrit before leukapheresis was positively correlated with the rate of leukocyte depletion. Low haematocrit means that plasma proportion is increased in the bloodstream, which makes the operator reduce the collected volume of each cycle, and the rate of leukocyte depletion decreases. The patients with lower HCT during apheresis are prone to anaemia, so it may improve the collection efficiency to transfuse the red blood cells. A higher HCT indicates a higher collection efficiency of WBC (CE_WBC_) (Fig. 3), but the CE_WBC_ is not significantly related with the overall survival (OS), because OS is not only influenced by CE_WBC_ but is mainly affected by the late chemotherapy. The HCT is not an independent prognostic factor in our study, and the overall survival of patients was affected by other factors. Reinhart reported an optimum transfusion threshold of 20–24% HCT which was stable for hospitalised patients^31^. The relatively high haematocrit may exacerbate the intravascular viscosity of HLL patients^32^, and low HCT could reduce vascular thrombosis, cardiovascular diseases and other complications^33,34^. For HLL patients, the HCT < 24% may lead to fewer complications and has relatively higher OS in our study.

To reduce the white blood cells, platelets and red blood cells also have varying degrees of reduction simultaneously^28^. During our leukapheresis, the blood components such as platelets and red blood cells are also lost and remained in the apheresis pipe and contaminated in the leukocytes. Among these decreased blood components, monocyte, neutrophil, and platelet decreased the most, suggesting that potential haemorrhage and infection should be taken into consideration. Leukapheresis was associated with clinically significant decreases in platelets and fibrinogen and prolonged clotting times in hyperleukocytic AML patients^35^. Clinical leukapheresis requires systemic blood circulation with long procedure time, which is difficult to tolerate for patients. In addition, it involves high cost for patients due to the low centrifugal separation purity and the need to often collect samples many times. So far, there is no precise separation scheme for leukocyte, it is an urgent problem to be solved clinically to realise the efficient separation of leukocytes. The technical aspects and efficacy of leukapheresis will be considered in the prophylaxis and management of the emergent demands.

In conclusion, hyperleukocytosis is of prognostic importance in several types of leukaemias, and the use of emergent leukapheresis is an imperative method. Our study suggested that the rate of leukocyte depletion was negatively correlated with WBC counts before leukapheresis and positively correlated with HCT pre-apheresis. Increase in haematocrit level will contribute to improved leukocyte depletion for the HLL patient during the procedure. It also exits the blood loss in leukapheresis, so the research and development of efficient white blood cell separation technology and clinical equipment will become the future direction of leukaemia treatment.

## Materials and Methods

### Patients

Between May 2010 and December 2016, a total number of 67 cases with newly diagnosed acute myeloid leukaemia (AML, M3 subtype excluded) admitted to our institution with an initial white blood cell (WBC) counts greater than 50 × 10^9^/l were scheduled to undergo leukapheresis. AML diagnosis was established on the basis of standard morphologic and cytochemical examinations of peripheral blood and marrow smears according to the French-American-British (FAB) and the World Health Organization (WHO) criteria^26,36,37^. The diagnostic tests included bone marrow biopsy, flow cytometry, fluorescence *in situ* hybridization (FISH), PCR and so on. The risk stratification for AML patients is mainly based on the European LeukaemiaNet (ELN) classification^26,38,39^. Both studies were conducted in line with the Declaration of Helsinki and approved by the institution’s Research Ethics Committee of Zhongnan Hospital. The patients obtained the informed consent form and data were collected from the electronic patient record.

There are no evidence-based guidelines for when to start leukapheresis, whether leukapheresis should be performed for HLL patients is determined by peripheral WBC counts and the state of hyperleukocytosis or inability to start induction chemotherapy immediately^40,41^. When patients with WBC counts above 50 × 10^9^/l present with respiratory failure, dizziness, headache, bone pain, lymphadenopathy, hepatomegaly, splenomegaly, tinnitus, blurred vision, somnolence, stupor, or delirium, they will undergo leukapheresis based on physician preferences^41^. Leukapheresis is not recommended for patients with thrombocytopenic coagulopathy due to elevated risk of bleeding. No patients had received chemotherapy before apheresis. The patients were placed in the haematological intensive care after admission to our hospital and underwent leukapheresis followed by treatment with hydroxylurea orally until WBC counts < 50 × 10^9^/l were achieved, and then received standard regularisation chemotherapy.

### Therapeutic Leukapheresis

The Fresenius COM.TEC machine runs with a continuous blood flow. At the start of the leukapheresis procedure, the donor’s sex, body weight, height, haematocrit level and white blood cell (WBC) counts were entered into the machine. The COM.TEC instrument parameters were set as follows: whole blood flow rate set at 30–60 ml/min, single cycle volume at 180 ml, the number of cycles was set based on the processed blood volume which is at least above the two-fold of total blood volume, anticoagulant/whole blood ratio = 1:9. The white blood cell (WBC) collection by the COM.TEC cell separator is controlled via the MNC collection computer software (version 4.0). Prophylactic calcium, 20 ml of 10% calcium gluconate orally or 500 ml normal saline plus 40 ml 10% calcium gluconate intravenous infusion, were administered to all patients. Dual-needle leukapheresis kits were used according to the manufacturer’s instructions (Fresenius Kabi, Germany).

We analyzed only the first collected information of the 67 patients, regardless of how many procedures were performed. Subsequent collected data was not included in our analysis to avoid data heterogeneity.

### Leukapheresis operational variables

Blood volume processed (BVP) was the volume of blood in the circulatory system of any individual. For all procedures, the separation time, total blood volume (TBV), BVP, collection capacity, flow rate and time were recorded in detail.

### Evaluation of the Leukapheresis efficiency

Peripheral blood samples (2 ml, ethylenediaminetetraacetic acid [EDTA]) were drawn from each patient (inlet line) prior to and two hours after completion of leukapheresis. Leukapheresis product samples with EDTA (2 ml) were obtained for laboratory analysis. Pre-leukapheresis and post-leukapheresis complete blood counts (CBC) and collected product were performed to determine the blood routine examination with an automated blood cell analyser Coulter STKS (Beckman Coulter, USA). Harvest product was further analysed for volume, the numbers of collected total nucleated cells.

The collection efficiencies (CE_WBC_) were calculated based on patient’s blood volume by the following formula^42,43^:

1 $$\begin{array}{c}{\bf{C}}{\bf{E}}\,({\bf{r}}{\bf{a}}{\bf{t}}{\bf{e}}\,{\bf{o}}{\bf{f}}\,{\bf{l}}{\bf{e}}{\bf{u}}{\bf{k}}{\bf{o}}{\bf{c}}{\bf{y}}{\bf{t}}{\bf{e}}\,{\bf{d}}{\bf{e}}{\bf{p}}{\bf{l}}{\bf{e}}{\bf{t}}{\bf{i}}{\bf{o}}{\bf{n}})\\ \,\,\,=\,\frac{{\rm{W}}{\rm{B}}{\rm{C}}\,{\rm{c}}{\rm{o}}{\rm{u}}{\rm{n}}{\rm{t}}{\rm{s}}\,{\rm{i}}{\rm{n}}\,{\rm{c}}{\rm{o}}{\rm{l}}{\rm{l}}{\rm{e}}{\rm{c}}{\rm{t}}{\rm{e}}{\rm{d}}\,{\rm{p}}{\rm{r}}{\rm{o}}{\rm{d}}{\rm{u}}{\rm{c}}{\rm{t}}\times {\rm{v}}{\rm{o}}{\rm{l}}{\rm{u}}{\rm{m}}{\rm{e}}\,{\rm{o}}{\rm{f}}\,{\rm{c}}{\rm{o}}{\rm{l}}{\rm{l}}{\rm{e}}{\rm{c}}{\rm{t}}{\rm{e}}{\rm{d}}\,{\rm{p}}{\rm{r}}{\rm{o}}{\rm{d}}{\rm{u}}{\rm{c}}{\rm{t}}\,({\rm{m}}{\rm{l}})}{{\rm{W}}{\rm{B}}{\rm{C}}\,{\rm{c}}{\rm{o}}{\rm{u}}{\rm{n}}{\rm{t}}{\rm{s}}\,{\rm{p}}{\rm{r}}{\rm{e}}-{\rm{a}}{\rm{p}}{\rm{h}}{\rm{e}}{\rm{r}}{\rm{e}}{\rm{s}}{\rm{i}}{\rm{s}}\times {\rm{t}}{\rm{o}}{\rm{t}}{\rm{a}}{\rm{l}}\,{\rm{b}}{\rm{l}}{\rm{o}}{\rm{o}}{\rm{d}}\,{\rm{v}}{\rm{o}}{\rm{l}}{\rm{u}}{\rm{m}}{\rm{e}}\,({\rm{m}}{\rm{l}})}\times 100{\rm{ \% }}\end{array}$$

In addition, the RBC and PLT contamination were also evaluated.

2 $$\begin{array}{c}{\bf{blood}}\,{\bf{cell}}\,{\bf{reduction}}\\ \,=\,{\rm{blood}}\,{\rm{cell}}\,{\rm{counts}}\,{\rm{before}}\,{\rm{leukocytapheresis}}-{\rm{blood}}\,{\rm{cell}}\,{\rm{counts}}\,{\rm{after}}\,{\rm{leukocytapheresis}}{\rm{.}}\end{array}$$

### Laboratory tests

The hepatic and renal function were tested by Olympus 5400 automatic biochemical analyser (Beckman, USA). The immunoglobulin was analysed by BN ProSpec^®^ automatic protein analyser (Siemens Healthineers).

### Statistical analysis

Statistical analysis was performed using the Statistical Package for the Social Sciences version 24.0 (IBM SPSS 24.0). Data was expressed as means ± standard deviations or median (range). The paired *t*-test was used to compare the results of laboratory test before and after leukocytopheresis. For most numerical variables, two groups were formed using the median as a cut-off value^44^. The clinical parameters were categorised according to the most discriminating cut-off values, lower than the median as a group, higher than the median for another set. The categorical variables were compared using the independent samples nonparametric Mann-Whitney U-test. Bivariate correlation analysis was performed to characterise the correlation between the rate of leukocyte depletion and clinical parameter by nonparametric Spearman test (r). The multivariate analysis of factors influencing the CE_WBC_ were determined by multiple linear regression model with backward stepwise variable selection. Multivariate survival analyses were performed to identify independent factors for overall survival. A stratified overall survival analysis was performed using the Kaplan-Meier method followed by the log-rank test and Cox regression model with forward stepwise^45^. All P values are two-sided, *p* < 0.05 was regarded as statistically significant.

### Data availability statement

All data generated or analysed during this study are included in this published article (and its Supplementary Information files).

## Electronic supplementary material

Supplemental Table S1

Supplementary Information
