# Supplementary material for: A hospital based retrospective study of factors influencing therapeutic leukapheresis in patients presenting with hyperleukocytic leukaemia
Source: Sci Rep. 2018 Jan 10;8:294. doi: 10.1038/s41598-017-17534-4 (PMC5762875; doi:10.1038/s41598-017-17534-4)
Supplement: Supplementary file 2 — Supplementary Information [file 41598_2017_17534_MOESM2_ESM.pdf]

A hospital based retrospective study of factors influencing therapeutic leukapheresis in patients presenting with hyperleukocytic leukaemia

Yanxia Jin[1], Shishang Guo[2], Qin Cui[1], Sichao Chen[1], Xiaoping Liu[1], Yongchang Wei[3, 5], Yunbao Pan[4, 5], Liang Tang[6], Tingting Huang[1], Hui Shen[1], Guanghui Xu[6], Xuelan Zuo[1], Shangqin Liu[1], Hui Xiao[1], Fei Chen[1], Fayun Gong[*, 6] & Fuling Zhou[*, 1, 3, 4]

Supplementary Information

Supplemental Figure S1 The normal distribution of WBC counts were shown pre-apheresis and post-apheresis.

Supplemental Figure S2 The survival rate of HLL patients was associated with ELN classification.

Supplemental Table S1 The additional patient information.

Supplemental Table S2 The median of factors pre-apheresis.

Supplemental Table S3 The association of clinical parameters with rate of leukocyte depletion in HLL Patients.

Supplemental Table S4 Bivariate correlation analysis between clinical parameters pre-apheresis and rate of leukocyte depletion in HLL Patients.

Supplemental Table S5 The correlation of each parameter before leukapheresis in HLL Patients.

Supplemental Table S6 The correlations between clinical parameters pre-apheresis and the presence of symptoms/failures caused by the hyperleukocytosis.

Supplemental Figure S1 The normal distribution of WBC counts were shown pre-apheresis and post-apheresis.

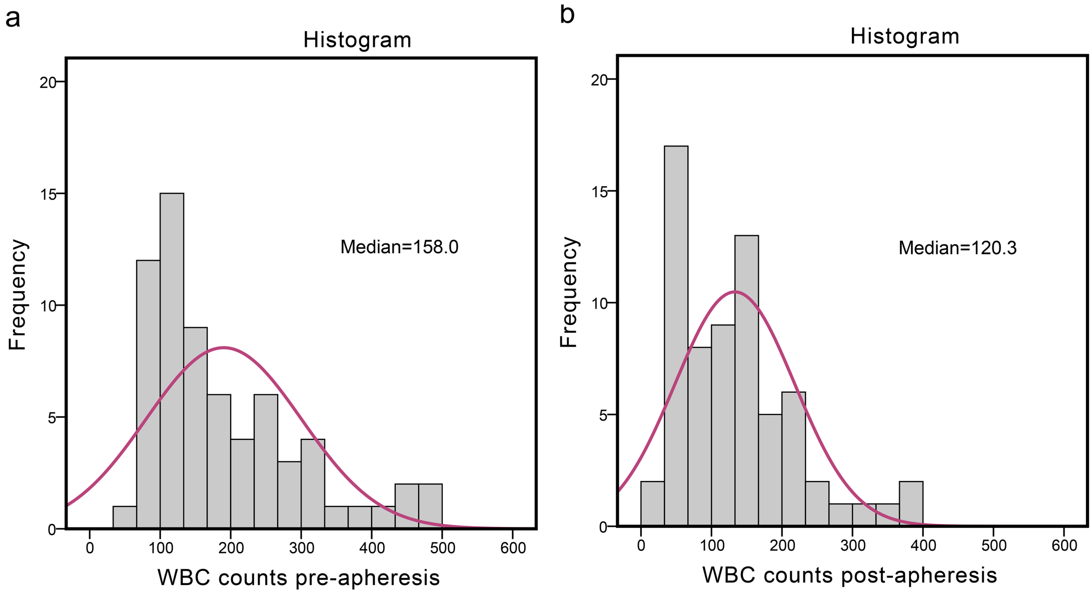

Supplemental Figure S2 The survival rate of HLL patients was associated with ELN classification.

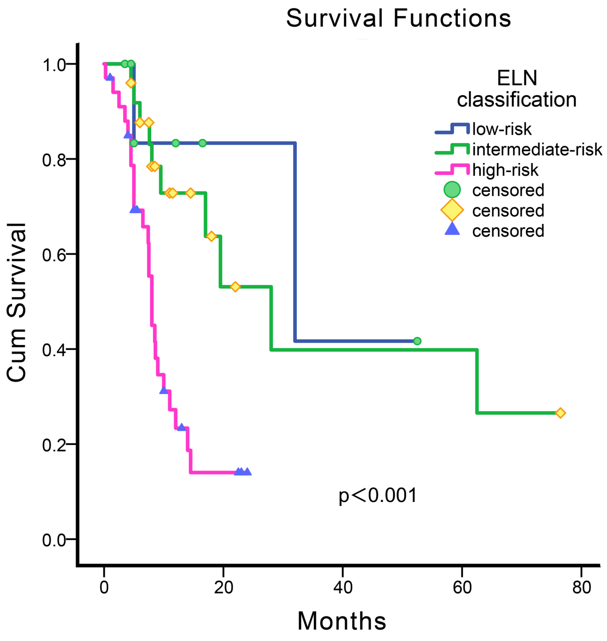

Supplemental Table S2 The median of factors pre-apheresis.

**Laboratory tests**		**median**
**Hepatorenal function**	AST (u/l)	26.00
	ALT (u/l)	24.00
	TBIL (μmol/l)	12.70
	BUN (mmol/l)	5.90
	CREA (μmol/l)	73.50
	UA (μmol/l)	394.00
**Electrolyte**	K (mmol/l)	3.64
	Na (mmol/l)	139.70
	Cl (mmol/l)	102.00
	Ca (mmol/l)	2.06
	P (mmol/l)	1.27
**Serum proteins**	TP (g/l)	61.10
	ALB (g/l)	35.80
	GLB (g/l)	18.50

Supplemental Table S3 The association of clinical parameters with rate of leukocyte depletion in HLL Patients.

**Items**		**Rate of leukocyte depletion (*p* value)**
**Baseline**	Age	**0.032**
	Weight	0.214
**Product**	Total blood volume	0.950
	Blood volume processed	0.065
	Product volume	0.254
**Blood routine before apheresis**	RBC	0.900
	WBC	**0.004**
	PLT	**＜0.001**
	MNC	**0.049**
	LY	0.067
	NEU	0.341
	HGB	**0.009**
	HCT	**0.001**
	MCH	0.498
	MCV	0.145
	MCHC	0.107
**Hepatorenal function before apheresis**	ALT	0.595
	AST	0.949
	TBIL	0.532
	BUN	0.242
	CREA	0.750
	UA	0.331
**Electrolyte before apheresis**	K	0.433
	Na	0.674
	Cl	**0.029**
	Ca	0.085
	P	0.167
**Serum proteins before apheresis**	TP	0.069
	ALB	0.140
	GLB	**0.034**

Analysed the median of each clinical parameters, lower than the value as a group, higher than the value for another set, then compared the difference of rate of leukocyte depletion between the two groups with nonparametric test (Mann-Whitney U test) in two independent samples.

The cutoff value for age was 55 years, WBC pre-apheresis was 100×10^9/l, PLT pre-apheresis was 100×10^9/l, HGB pre-apheresis was 100 g/l, HCT pre-apheresis was 24%, LY pre-apheresis was 40.0×10^9/l. Other variables are grouped with the median.

Supplemental Table S4 Bivariate correlation analysis between clinical parameters pre-apheresis and rate of leukocyte depletion in HLL Patients.

**Item**	**r**	***p* value**
WBC	-0.477	＜0.001
PLT	-0.488	＜0.001
MNC	0.376	0.018
NEU	-0.361	0.022
HCT	0.489	＜0.001
HGB	0.338	0.006
ALB	-0.363	0.014
MCV	0.263	0.031
MCHC	-0.276	0.024
K	-0.302	0.035
P	-0.304	0.033

Supplemental Table S5 The correlation of each parameter before leukapheresis in HLL patients.

**Item**		**WBC**	**PLT**	**MO**	**HGB**	**HCT**
Rate of WBC depletion	r	-0.477	-0.488	0.376	0.338	0.489
	p value	**＜0.001**	**＜0.001**	**0.018**	**0.006**	**＜0.001**
WBC	r		0.225	0.117	-0.216	-0.286
	p value		0.067	0.477	0.081	**0.019**
PLT	r			-0.578	0.070	-0.221
	p value			**＜0.001**	0.576	0.072
MO	r				0.207	0.516
	p value				0.212	**0.001**
HGB	r					-0.806
	p value					**＜0.001**

r: Correlation coefficient.

Supplemental Table S6 The correlations between clinical parameters pre-apheresis and the presence of symptoms/failures caused by the hyperleukocytosis.

Correlation analysis		ALT	AST	TBIL	TP	ALB	GLB	BUN	CREA	UA
WBC	Pearson Correlation	.025	.172	-.021	-.076	.053	.055	**.411****	**.320***	**.570****
	Sig. (2-tailed)	.867	.248	.892	.620	.730	.718	**.004**	**.028**	**.000**
Survival	Pearson Correlation	.108	-.052	-.018	-.145	-.196	-.063	-.026	.043	.127
	Sig. (2-tailed)	.471	.731	.905	.341	.198	.680	.863	.774	.391
Sex	Pearson Correlation	**-.397****	**-.401****	-.218	**.507****	**.416****	.198	-.237	**-.441****	**-.471****
	Sig. (2-tailed)	**.006**	**.005**	.151	**.000**	**.004**	.192	.105	**.002**	**.001**
Age	Pearson Correlation	-.188	**-.346***	-.162	.054	-.167	.145	-.132	-.011	-.231
	Sig. (2-tailed)	.206	**.017**	.287	.727	.273	.341	.370	.941	.114
Height	Pearson Correlation	.200	-.104	.200	-.221	-.092	-.147	-.036	**.398****	.201
	Sig. (2-tailed)	.177	.486	.189	.144	.550	.336	.808	**.006**	.171
Weight	Pearson Correlation	**.292***	.049	**.494****	-.178	-.124	**-.363***	-.002	**.442****	**.292***
	Sig. (2-tailed)	**.046**	.742	**.001**	.243	.416	**.014**	.990	**.002**	**.044**
RBC	Pearson Correlation	-.106	-.262	.076	**.317***	**.526****	-.008	-.019	.032	-.211
	Sig. (2-tailed)	.480	.075	.618	**.034**	**.000**	.957	.899	.830	.151
PLT	Pearson Correlation	-.110	-.235	-.022	**.353***	**.543****	-.105	.211	-.002	-.030
	Sig. (2-tailed)	.463	.112	.885	**.017**	**.000**	.493	.150	.990	.839
MO	Pearson Correlation	-.083	.064	-.009	-.156	-.174	**.375***	-.262	.162	**.438***
	Sig. (2-tailed)	.651	.726	.960	.402	.349	**.038**	.141	.367	**.011**
LY	Pearson Correlation	-.230	-.031	-.106	-.064	-.164	**.550****	-.160	.029	**.510****
	Sig. (2-tailed)	.214	.868	.578	.738	.387	**.002**	.383	.873	**.003**
NE	Pearson Correlation	-.302	-.041	**-.515****	-.093	-.013	**.567****	**.517****	.060	**.516****
	Sig. (2-tailed)	.105	.831	**.004**	.633	.948	**.001**	**.003**	.747	**.003**
HCT	Pearson Correlation	.157	.044	-.137	**-.407****	**-.336***	.084	-.146	.063	-.046
	Sig. (2-tailed)	.292	.771	.369	**.006**	**.024**	.585	.323	.674	.758
HGB	Pearson Correlation	.095	-.071	-.039	-.255	-.176	.013	-.179	.001	-.107
	Sig. (2-tailed)	.529	.640	.804	.095	.252	.935	.229	.996	.473

*$p < 0.05$, **$p < 0.01$.
